# The pathogenesis of endemic fluorosis: Research progress in the last 5 years

**DOI:** 10.1111/jcmm.14185

**Published:** 2019-02-19

**Authors:** Wei Wei, Shujuan Pang, Dianjun Sun

**Affiliations:** ^1^ Key Lab of Etiology and Epidemiology, Education Bureau of Heilongjiang Province & Ministry of Health, Center for Endemic Disease Control, Chinese Center for Disease Control and Prevention Harbin Medical University Harbin China; ^2^ Institution of Environmentally Related Diseases Harbin Medical University Harbin China

**Keywords:** apoptosis, fluoride, pathogenesis, research progress, signalling pathways

## Abstract

Fluorine is one of the trace elements necessary for health. It has many physiological functions, and participates in normal metabolism. However, fluorine has paradoxical effects on the body. Many studies have shown that tissues and organs of humans and animals appear to suffer different degrees of damage after long‐term direct or indirect exposure to more fluoride than required to meet the physiological demand. Although the aetiology of endemic fluorosis is clear, its specific pathogenesis is inconclusive. In the past 5 years, many researchers have conducted in‐depth studies into the pathogenesis of endemic fluorosis. Research in the areas of fluoride‐induced stress pathways, signalling pathways and apoptosis has provided further extensive knowledge at the molecular and genetic level. In this article, we summarize the main results.

## 1 INTRODUCTION

Fluorine is widely dispersed in nature, almost entirely in the form of fluorides. In some locations, such as some areas of China, India, and Bengal, the drinking water contains dangerously high levels of fluoride (>1.2 mg/L), leading to serious health problems. Fluoride can be present in tea leaves, following absorption from soil and water. The mature leaves contain as much as ten to 20 times the fluoride levels of young leaves from the same plant.^1^ When the tea leaves are soaked in water, the fluoride in tea leaves can also be taken up by the body. In some areas where brick tea is heavily consumed, the intake of fluoride via tea can be several times greater. In addition, in some provinces of China, there is the habit of burning bituminous coal, which has a high fluoride content, to cook, and dry food. With exposure to atmospheric pollution derived from burning these fossil fuels over a long period, fluoride absorbed through the respiratory and digestive tracts also increases significantly. Ingestion of excessive amounts of fluoride can cause damage to various bodily systems or organs. The major clinical manifestations are presented as dental fluorosis, skeletal fluorosis and other symptoms in non‐skeletal tissue caused by excessive accumulation of fluoride.

**Figure 1:**
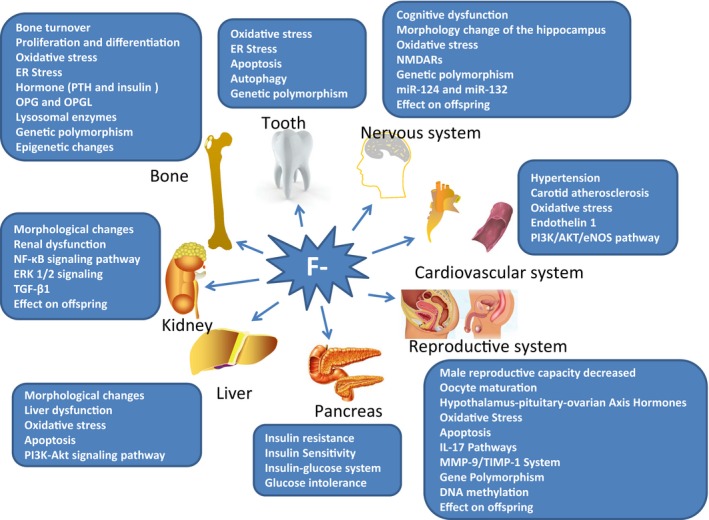
The key words for pathogenesis of endemic fluorosis in the last 5 years. From the perspective of the effects of fluoride on different tissues and organs of the body, research work from the last five years has mainly focused on effects at the molecular and genetic levels, such as fluoride‐induced stress pathways, signalling pathways and apoptosis

Since the main manifestations of fluorosis are dental and skeletal fluorosis, researchers have long focused on the pathology of bone and tooth tissues. In the past 5 years, new progress has been made in research into the pathogenesis of dental and skeletal fluorosis. In addition, researchers have begun to pay more attention to the effects of fluoride on other organs and systems in the body. The largest body of research in recent years has investigated the mechanism of action of fluoride on non‐skeletal tissue. The Conference of the International Society for Fluoride Research is organized by the International Society for Fluoride Research and represents the latest advances in international fluoride research. The 33rd Conference of the International Society for Fluoride Research, held in India in 2016, focused on the pathogenesis of fluorosis at the molecular and genetic level. It not only explores the molecular mechanism of fluoride action in bone tissue damage, but also the toxic effects of fluoride on non‐skeletal tissues, such as the nervous system, cardiovascular system, liver, kidney, reproductive system, thyroid and the progeny effect of fluoride. Therefore, in this article, we will review research work from the last five years from the perspective of the effects of fluoride on different tissues and organs of the body. The key words for pathogenesis of endemic fluorosis in the last 5 years are shown in Figure 1.

## 2 MECHANISM OF ACTION OF FLUORIDE ON BONE AND TOOTH TISSUES

### 2.1 The pathogenesis of dental fluorosis

Dental fluorosis is the earliest specific clinical manifestation of endemic fluorosis. The pathological changes mainly occur in enamel, but dentin and cementum are also involved. In recent years, research into the pathogenesis mainly focused on fluoride interference with protein secretion of ameloblasts, resulting in amelogenin hydrolysis and removal delay, and differences in susceptibility to fluoride due to individual genotypes.

#### 2.1.1 Effects of fluoride on the signalling pathway of ameloblasts

Development of the tooth germ involves the process of ameloblast and odontoblast differentiation, leading to tooth hard tissue formation. Fluoride may cause disordered protein synthesis by affecting the function of the endoplasmic reticulum in ameloblasts. Recent studies found that fluoride can cause glucose‐regulated protein78 up‐regulation in ameloblasts,^2^ and activate inositol‐requiring kinase 1α and transcription factor 6 pathway in unfolded protein reactions,^3^ thereby interfering with the secretory function of ameloblasts, resulting in the development of dental fluorosis. Oxidative stress is also related to the occurrence of dental fluorosis. Excessive fluoride can induce oxidative stress in ameloblasts,^4^ and the fluoride‐induced reactive oxygen species (ROS) production causes oxidative damage to mitochondria and DNA,^5^ leading to activation of SIRT1/autophagy via ROS‐mediated JNK signalling. In addition, excessive fluoride can induce apoptosis of ameloblasts. The expression of and CHOP^2^ in ameloblasts increases with the increase of fluoride concentration. It is speculated that apoptosis induced by the endoplasmic reticulum stress pathway may play a role in the occurrence of dental fluorosis. High‐fluoride partially activates the FasL,^6^ p‐ERK and p‐JNK signalling pathways^7^ of ameloblasts, leading to increased expression of apoptotic genes, indicating that oxidative stress is closely associated with apoptosis in dental fluorosis. Fluoride can also induce apoptosis by increasing the phagocytic activity of mature ameloblasts, and the Bcl‐2 signalling pathway is involved in this process.^8^ Furthermore, there is evidence that autophagy is involved in dental fluorosis.^4^ One study^9^ observed that fluoride increased expression of Beclin1, which is required for autophagosome formation, and decreased the expression of mTOR, an autophagy‐related complex, indicating that autophagy is involved in dental fluorosis.

#### 2.1.2 Relationship between dental fluorosis and genetic polymorphism

In recent years, different individuals with genotypes susceptible to fluoride began to attract the attention of researchers. In the same population with the same fluoride exposure levels, there is a large difference in the extent of dental fluorosis between individuals, which may be related to genetic background and individual susceptibility to fluoride. The site Alu I CT + TT of the calcitonin receptor (CTR) is a susceptible genotype in populations with coal‐burning type fluorosis. The polymorphism of the *CTR* gene may affect ion metabolism during tooth mineralization, resulting in differences in the occurrence of dental fluorosis at the same fluoride level.^10^ Some studies have also shown a relationship between different loci of the same gene and dental fluorosis. Ten years ago, a case‐control study showed a possible association between polymorphisms (Pvu II and Rsa I) in the *COL1A2* gene and dental fluorosis in high fluoride‐exposed populations.^11^ However, recently, a cross‐sectional study showed that the presence of an A/C polymorphism in the *COL1A2* gene was not associated with the severity of dental fluorosis in drinking water‐type fluorosis.^12^ Another study showed that the polymorphisms in the enamel matrix genes *AMBN*, *TFIP11*, and *TUFT1* were associated with dental fluorosis.^13^

### 2.2 The pathogenesis of skeletal fluorosis

Skeletal fluorosis includes osteosclerosis, osteomalacia, osteoporosis, ossification of peri‐osseous soft tissue and degenerative changes of cartilage and joints. Active osteogenesis and accelerated bone turnover are important features of skeletal fluorosis progression and the pathological basis of the diversity of osteogenic lesions. In recent years, studies of the pathogenesis of skeletal fluorosis have focused on the various cell regulatory mechanisms by which fluoride affects the process of bone turnover.

#### 2.2.1 Effects of fluoride on osteoblasts

Bone lesions caused by fluoride are complex and diverse, and affect a variety of cells involved in bone turnover. Among them, the aberrant activation of osteoblasts in the early stage plays a critical role. In recent years, a series of studies on the proliferation and differentiation of osteoblasts stimulated by fluoride have found that the BMP/Smad signalling pathway^14^ and the Wnt and notch pathways^15^ in osteoblasts may be involved. In addition, skeletal fluorosis is closely related to endoplasmic reticulum stress and oxidative stress. Fluoride induces the endoplasmic reticulum stress response of osteoblasts, then endoplasmic reticulum stress response unfolded proteins are involved in osteoblast differentiation.^16^, ^17^ The protein kinase RNA (PKR)‐like ER kinase (PERK) pathway is associated with fluoride‐induced bone formation and bone resorption.^18^ Another study shows that fluoride‐induced osteoblast apoptosis may be regulated through ROS levels and mitochondrial membrane potentials.^19^ In addition, fluoride can affect hormone levels, thereby contributing to active bone turnover. Studies have shown that increased secretion of parathyroid hormone (PTH) plays an important role in the pathogenesis of fluoride‐induced osteogenesis and accelerated bone turnover,^20^ and that PTH participates in the process of fluoride modulation of SOST/Sclerostin and RANKL expression.^21^ Another study confirmed that insulin not only stimulates the activity of osteoblasts, but also enhances the role of fluoride in stimulating osteoblast activity.^22^

#### 2.2.2 Effect of fluoride on osteoclasts

One of the pathological changes of skeletal fluorosis is the development of osteoporosis and osteopetrosis. In the development of skeletal fluorosis, the active function and absorption of osteoclasts plays an important role in the pathogenesis of osteoporosis. One report elucidated that the transforming growth factor (TGF) beta receptor 1/Smad3 pathway participated in the mechanism of biphasic modulation of osteoclast mode, regulated by fluoride.^23^ RANKL is necessary for osteoclast formation. Excessive fluoride exposure can stimulate osteoblasts to secrete RANKL,^24^ and the effect of fluoride on osteoclasts differs under different concentrations of RANKL. Fluoride also regulates the expression of nuclear factor of active T cells (NFAT) c1 in osteoclasts. In vitro studies have shown that fluoride can reduce the activity of osteoclasts by inhibiting the expression of *NFATc1* and downstream genes,^25^ but the specific mechanism remains to be further studied. The ratio of osteoprotegerin ligand (OPGL) to osteoprotegerin (OPG) can accurately regulate the balance between bone resorption and synthesis. In a study of fluorosis in rats, it was found that OPG and OPGL may play important roles in skeletal fluorosis, and fluoride may enhance osteoclast formation and induce osteoblastic destruction.^23^

#### 2.2.3 Effect of fluoride on chondrocytes and cellular matrix in bone and cartilage

Excessive fluoride interferes with bone metabolism partly by affecting the extracellular matrix of bone tissue. Osteoblasts are active but form immature woven bone in fluorosis. The structure and arrangement of collagen obviously differ from those of mature lamellar bone. Collagen is one of the important components of bone and cartilage tissue, with type I collagen being the main type of collagen in bone tissue, while in cartilage the main type is type II collagen. Excessive fluoride can cause metabolic abnormalities of bone and cartilage collagen. Studies have shown that excess fluoride can cause type I collagen disorder, leading to changes in the ultrastructure of bone tissue.^26^ Another study showed that NaF decreases the secretion of chondrocyte type II collagen in primary cultured rat chondrocytes, possibly through the down‐regulation of HIF‐1α via the Sox9 pathway.^27^ Excessive fluoride affects the function of osteoclasts and promotes the secretion of some lysosomal enzymes by osteoclasts, mainly acid and matrix metalloproteinases (MMPs) which promote the degradation of matrix and accelerate the process of bone turnover. In a study of the effects of fluoride on RANKL‐induced osteoclast differentiation, we found that MMP9 and cathepsin K can be used as indicators of changes in bone resorption activity and fluoride exposure.^28^

#### 2.2.4 Genetic polymorphisms, Epigenetic changes and skeletal fluorosis

The role of individual differences in the pathogenesis of skeletal fluorosis has drawn the attention of researchers in recent years. It was noticed that even in cases involving similar doses of fluoride exposure, different ethnic groups showed differences in the incidence of skeletal fluorosis,^29^ suggesting that human genes play an important role in the pathogenesis of endemic fluorosis. A population‐based study showed that polymorphism of the glutathione S‐transfected P1 rs1695 gene was associated with the prevalence of tea‐type skeletal fluorosis.^30^ In the Tibetan population, those with the G allele have a reduced risk of skeletal fluorosis. Another study showed that polymorphism of the myeloperoxidase gene was related to fluorosis in adults living in the coal‐burning endemic fluorosis area in Guizhou, China.^31^ These results suggest that genetic polymorphism may play an important role in the pathogenesis of fluorosis.

The role of histone modification in the pathogenesis of skeletal fluorosis has been investigated. The result shows that fluoride‐induced hyper H3K9 tri‐methylation‐mediated repression of TGFBR2 and Smad3 was related to the development of skeletal fluorosis.^32^ Preliminary exploration of the relationship between the epigenetic changes and skeletal fluorosis provides a new perspective for the study of the pathogenesis of skeletal fluorosis.

## 3 MECHANISM OF ACTION OF FLUORIDE ON NON‐SKELETAL TISSUES

Fluorosis can cause varying degrees of extensive non‐skeletal damage, with obvious diversity, and the specific mechanism varies. The common features are mostly degenerative changes in parenchymal cells without significant inflammatory response. In addition to the effects of super‐large doses, which can lead to obvious cell necrosis, in most cases increased apoptosis is observed. Most recent research suggests that the main regulatory mechanisms of the body, such as metabolism, stress and apoptosis, may all be changed to some extent under the influence of a certain concentration of fluoride. Excessive fluoride can cause oxidative stress and promote apoptosis of non‐bone tissue which is the current focus of research.

### 3.1 Effect of fluoride on the nervous system

Fluoride, like other halogen elements, can penetrate into the brain through the blood‐brain barrier. In recent years, the action of fluoride on the nervous system has attracted attention. Exposure to high levels of fluoride in water was found to be significantly associated with reduced levels of intelligence in children.^33^ Catechol‐O‐methyltransferase (*COMT*) gene polymorphisms may be related to the IQ of children exposed to fluoride in drinking water.^34^ Another study showed that high fluoride exposure may be a risk factor for cognitive dysfunction of the elderly over the age of 60 in drinking water‐type fluorosis areas where drinking water‐type fluorosis is endemic.^35^ Based on the results of the population survey, animal experiments in recent years have also focused on the study of the hippocampus which is responsible for learning and memory functions, since it is known that fluoride can damage the morphology of the hippocampus.^36^, ^37^ Fluoride also significantly changed hippocampal structural parameters of offspring after mothers were exposed to water fluorosis.^38^ Excessive fluoride can lead to increased production of nitric oxide and JNK signalling pathway activation,^39^ cerebral cortex nerve cell and synaptic redistribution,^40^ and abnormal accumulation of intracellular calcium.^41^ Sodium fluoride induced oxidative stress and also behavioural alteration in rat, and decreased levels of vitamin A can be used as a marker in fluoride‐induced toxicity studies.^42^ A recent study^43^ showed that neuronal destruction and synaptic injury caused by chronic fluorosis involve excitotoxicity. In addition, perinatal fluoride exposure can impair learning and memory in mouse offspring, possibly partly as a result of enhanced levels of miR‐124 and miR‐132 and alterations of their target genes.^44^ Another article^45^ showed that maternal fluoride exposure during gestation and lactation can influence the learning, memory ability and glutamate receptor expressions of the offspring.

### 3.2 Effects of fluoride on the cardiovascular system

The vascular wall is rich in collagen fibres, and collagen fibres are one of the major sites of action of fluoride. An epidemiological study in an endemic fluorosis area showed that excess fluoride intake has a certain connection with the incidence of adult hypertension, carotid atherosclerosis and the degree of disease lesions. The mechanism may be related to elevated levels of endothelin 1 in plasma caused by fluoride^46^ and leads to inflammation of the oxidative stress system and endothelial activation.^47^ The PI3K/AKT/eNOS pathway also plays a crucial role in the reduced expression of NO caused by excessive fluoride exposure.^48^ Based on these results, we speculated that fluoride may be involved in cardiovascular disease by causing damage to the vascular wall and myocardial injury, but its specific mechanism needs further study.

### 3.3 Effects of fluoride on the liver and kidney

Both liver and kidneys play important roles in the metabolism of fluoride in the body. Excessive intake of fluoride can affect liver function and kidney function, and cause pathological changes in liver and kidney tissues. In recent years, many scholars have explored the effects of fluoride on the liver and kidneys.

Fluoride has hepatotoxic effects. Fluoride can cause mouse liver dysfunction,^49^ induce morphological changes,^50^, ^51^ significantly increase hepatocyte apoptosis, promote the relative expression of caspase‐3 and caspase‐9 proteins and cause DNA damage^51^, ^52^ in the liver. Fluoride also can disturb lipid metabolism,^53^ cause the liver oxidative stress response,^52^, ^54^ and activate the PI3K‐Akt signalling pathway^55^ to participate in the pathogenesis of liver injury caused by fluorosis.

Renal pathological studies^56^, ^57^ have shown varying degrees of fluoride‐associated damage to the architecture of tubular epithelia, endothelial cells and of the mesangial cells of the renal glomerulus. Excessive intake of fluoride has been shown to alter the renal function parameters, and oxidative stress^50^, ^58^ and the NF‐κB signalling pathway^59^ plays an important role in the development of renal damage, which may eventually result in renal histopathological lesions and inflammatory responses. Fluoride can also cause renal cell injury by reducing the expression of extracellular signal‐regulated kinase (ERK) 1/2 in renal tissues,^60^ activating the M2 macrophage‐TGF‐β1‐fibroblast/myofibroblast‐collagen synthesis pathway.^61^ In addition, in female mice, intake of fluoride has a certain influence on the kidney function of their offspring.^57^

### 3.4 Effects of fluoride on the reproductive system

Excessive fluoride has damaging effects on the reproductive system in both men and women. Over the past decade, the effects of fluoride on the reproductive system have been the focus of much attention from researchers. Excessive fluoride exposure will affect the formation of male sperm and function of the reproductive endocrine system, resulting in decreased male reproductive capacity. Animal experiments show that fluoride reduces sperm motility, capacitation and the acrosome reaction leading to poor fertilization and suppressed embryonic development^62^; it also can significantly increase sperm mitochondrial DNA copy number, and reduce nuclear DNA integrity.^63^ A study indicated that fluoride exposure aggravates the degree of reproductive toxicity, such as sperm density, motility, viability and morphology as well as the testicular biochemical parameters in diabetic mice.^64^ Fluorosis‐induced spermatogenic cell apoptosis induced through the oxidative stress‐mediated JNK and ERK signalling pathways, and antioxidants such as VE or lycopene can reduce these reactive oxygen species‐induced toxic effects.^65^ Another study provided evidence that fluoride exposure up‐regulated the expression of the IL‐17 signalling pathway of spermatogenesis in the testicles of male mice by influencing many signalling pathways and genes, and that the PI3‐kinase/AKT, MAPKs and cytokines in the TGF‐β family contributed to this process.^66^ Excessive fluoride intake also has an influence on the reproductive system of the offspring of affected males, causing changes such as vacuolar dystrophy of epididymal epithelial cells, vacuolar dystrophy of linear seminal cells and necrosis.^67^

Fluoride also has adverse effects on the female reproductive system. A study^68^ on 18 to 48‐year‐old women living in fluorosis areas, fluoride exposure was found to affect the hypothalamus‐pituitary‐ovary axis hormone secretion. Animal experiments show that sodium fluoride exposure changes the histological structure of uterine tissue,^69^ alters ovarian morphology and apoptosis,^70^ impairs the maturation capacity of porcine oocytes,^71^ and hampers their development and fertilization.^72^ Another study provided compelling evidence that excessive fluoride intake can reduce the development potential of oocytes by inducing oxidative stress and apoptosis in the ovary of affected animals.^73^ In addition, fluoride can disturb DNA methylation of neuronatin (*NNAT*) and reduce oocyte quality by impairing glucose transport in porcine oocytes,^74^ as well as causing disturbance of the MMP‐9/tissue inhibitor of metalloproteinase‐1 system,^69^ all of which may be involved in fluoride‐induced reproductive dysfunction in females.

### 3.5 Relationship between fluoride and diabetes

One report^75^ studied the relationship between fluoride and diabetes in 22 states in the USA, and found that fluoride was significantly and positively associated with increases in both the incidence and prevalence of diabetes from 2005 to 2010 when accounting for per capita consumption of tap water. Another epidemiological study^76^ found that higher concentrations of fluoride in drinking water were associated with a higher incidence of childhood‐onset type 1 diabetes in the Newfoundland and Labrador provinces of Canada. Although there are several limitations to these studies, including the fact that diabetes most likely has a multifactorial aetiology, even including epigenetic processes, and the difficulty of unequivocally stating that these results are the specific consequences of water fluoridation, these studies provided new data concerning the role of fluoride on rates of diabetes. More comparable and more extensive analyses should be completed in other countries and areas to replicate the findings presented here. In the past 5 years, some investigations have also been carried out into the effects of fluoride on diabetic animals. Animal experiments showed that fluoride exposure increased insulin sensitivity in experimental diabetes,^77^ altered glucose homeostasis and led to insulin resistance.^78^ Another study^79^ observed alterations in muscle proteins related to glucose homeostasis in rats treated with fluoride.

**Figure 2:**
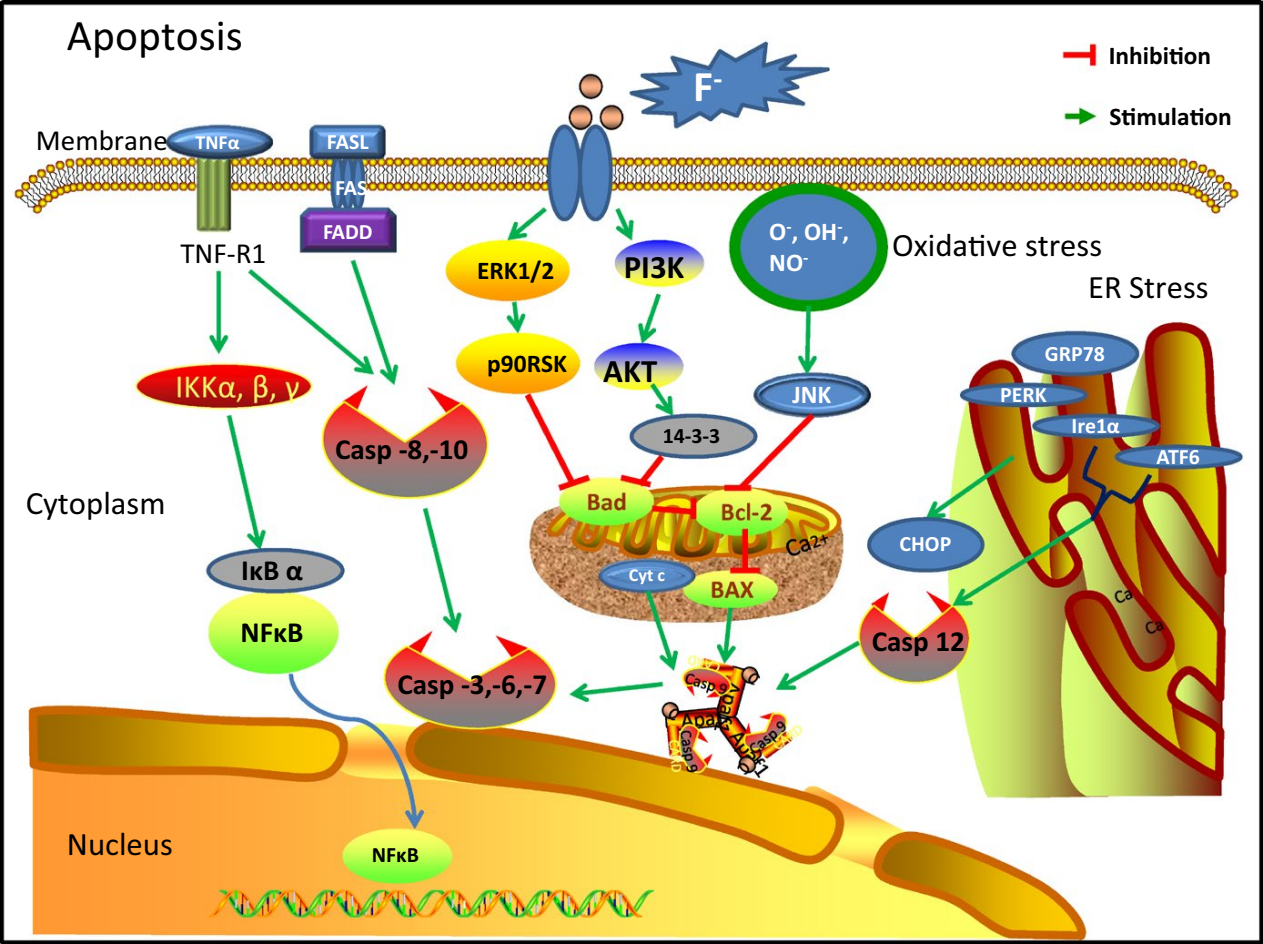
Common alterations in fluorosis‐apoptosis. Caspases, a family of cysteine proteases, are the central regulators of apoptosis. FasL can activate initiator caspases (Pro‐caspase 8 and 10), then cleave and activate the effector caspases 3, 6 and 7, leading to apoptosis. Fluoride exposure can activate these signalling pathways and induce apoptosis. In addition, excessive fluoride induces stress pathways such as oxidative stress and endoplasmic reticulum stress, thus promoting apoptosis. Many signalling pathways such as Erk1/2 and PI3K/Akt induce anti‐apoptotic Bcl‐2 family members. These Bcl‐2 family members protect the integrity of mitochondria, preventing Cytochrome C release and the subsequent activation of caspase‐9. TNF‐α may activate both pro‐apoptotic and anti‐apoptotic pathways. TNF‐α can induce apoptosis by activating caspase 8 and 10, but can also inhibit apoptosis via NF‐κB. Fluoride exposure can inhibit these survival signalling pathways

**Figure 3:**
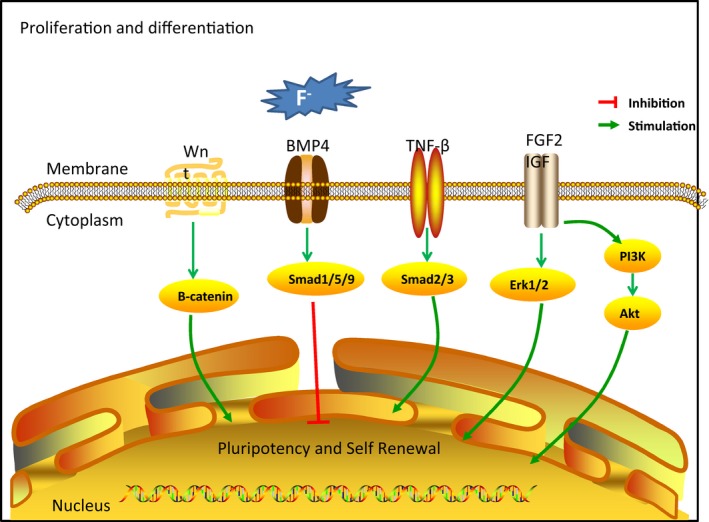
Common alterations in fluorosis‐proliferation and differentiation. The role of fluoride in cell proliferation and differentiation is the focus of research into the pathogenesis of skeletal fluorosis. The BMP/Smad and Wnt signalling pathways play important roles in the viability and differentiation of osteoblasts. TGFβ receptor 1‐smad3 signalling participates in the mechanism of biphasic modulation of osteoclast mode, regulated by fluoride. In addition, the FGF signalling pathway, which activates Akt and Erk1/2pathways, is responsible for the balance between cell proliferation and differentiation

**Figure 4:**
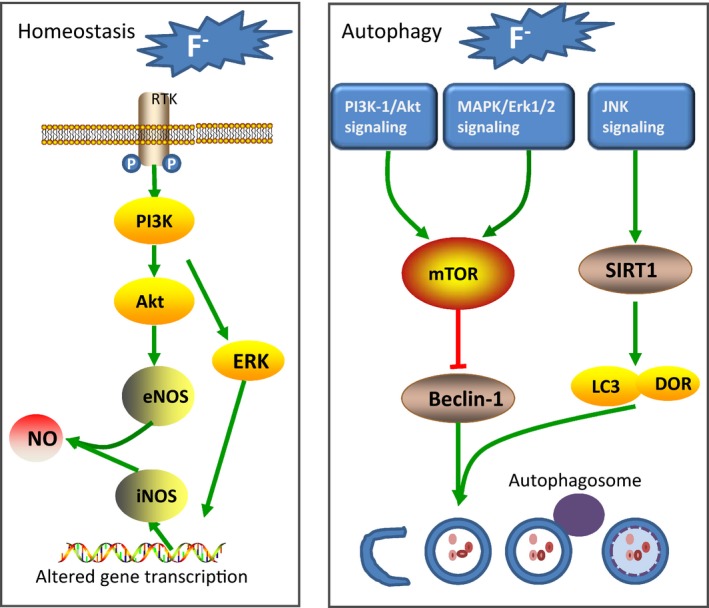
Common alterations in fluorosis‐homeostasis & autophagy. (a) NO is an important cellular signalling molecule. It helps modulate vascular tone, insulin secretion, airway tone and peristalsis, and is involved in angiogenesis and neural development. The PI3K/AKT/eNOS pathway plays a crucial role in homeostasis. Excessive fluoride exposure causes reduced expression of NO, which leads to damage of tissues and organs. (b) The kinase mTOR is a critical regulator of autophagy induction, with activated mTOR (PI3K‐1/Akt and MAPK/Erk1/2 signalling) suppressing autophagy. Fluoride can inhibit these pathways, so may lead to the development of autophagy. Besides, excessive fluoride exposure activates SIRT1/autophagy via JNK signalling

In summary, over the past 5 years there has been much progress in understanding of the pathogenesis of endemic fluorosis. In the field of fluoride‐induced stress pathways, signalling pathways and apoptosis, in‐depth understanding of its effects at the molecular (Summarized in Figures 2, 3, 4) and genetic level^80^ has increased. In addition, some research found that a number of signalling pathways involved in fluoride regulation of cell differentiation and function are damaged in skeletal tissue. These findings open up new perspectives on non‐skeletal fluorosis, and have provided new ideas for further molecular research into the mechanism of fluorosis. Although the aetiology of endemic fluorosis is clear, its specific pathogenesis is inconclusive. The exact pathogenesis remains to be further studied.

In the future, non‐skeletal tissue damage is still an area worth exploring. Epidemiological investigations have confirmed the effects of fluoride on some systems, such as the cardiovascular system, but further research on the pathogenesis is needed. In addition, there is no clear population survey of the relationship between fluoride and diabetes, which requires rigorous and large‐scale epidemiological investigations to further prove. Furthermore, the influence of fluorine on offspring is also an interesting research direction. One of the weaknesses in previous studies was that the dose of fluoride used in animal and cell experiments was generally too high (See Supporting Information for more details of the fluoride dose used in animal and cell experiments). Consequently, in future, the focus of such research should be: First, is this type of damage seen in patients with fluorosis, within the range of doses to which the human body may be exposed? Second, if it occurs in the human body, is it early primary damage or a late secondary or concurrent change? Again, in experimental studies, using only the high doses of fluoride in order to cause damage to certain tissues, organs or cultured cells would not be acceptable; the changes in the tissues and cells at a range of fluoride doses should be observed. Most importantly, the characteristic change is not simply cell apoptosis, necrosis and inhibition of function. Just as the characteristic change in skeletal fluorosis is that fluoride affects the process of bone turnover, it is necessary to distinguish which changes are specific to or characteristic of fluoride, and which are only the general toxic effects of high doses of poison. The use of gene chip technology and proteomics technology should also be considered in order to find breakthroughs. It is also necessary to conduct in‐depth study of the different mechanisms of fluoride in people with different genetic backgrounds and people with different types of fluorosis (such as drinking water type, coal‐burning type and brick‐tea type of endemic fluorosis).

## CONFLICTS OF INTEREST

The authors confirm that there are no conflicts of interest.
