# Supplementary material for: The pathogenesis of endemic fluorosis: Research progress in the last 5 years
Source: J Cell Mol Med. 2019 Feb 19;23(4):2333–42. doi: 10.1111/jcmm.14185 (PMC6433665; doi:10.1111/jcmm.14185)
Supplement: Supplementary file 1 [file JCMM-23-2333-s001.doc]

| **Table 1. Summary of epidemiological studies on the pathogenesis of fluorosis in five years** | | | | | | | | | | |
| --- | --- | --- | --- | --- | --- | --- | --- | --- | --- | --- |
| **Sample source** |  | **Research methods** |  | **Age at Baseline** |  | **Fluorosis type / F- concentration** |  | **Results** |  | **References** |
|  |  |  |  | **(Years)** |  |  |  |  |  |  |
| Blood and urine |  | Case-control study |  | Dental fluorosis : 8 – 12 |  | Coal-burning type fluorosis |  | Genotypes of 538-540 delGGA- /-and (986C T) CT in AMBN may interact with urinary fluoride (≥0. 95 mg / L) in the incidence of coal-burning fluorosis. |  | Jiang M et al. 2014 |
|  |  |  |  | Skeletal fluorosis : |  |  |  |  |  |  |
|  |  |  |  | Case group (45.00 ± 12.53) |  |  |  |  |  |  |
|  |  |  |  | Control group (43.00 ± 14.22) |  |  |  |  |  |  |
|  |  |  |  |  |  |  |  |  |  |  |
| Blood |  | Case-control study |  | Dental fluorosis : 8 – 12 |  | Coal-burning type fluorosis |  | The Alu I polymorphism of the CTR gene may be one of the genetic components associated with fluorosis. |  | Jiang M et.al 2015 |
|  |  |  |  | Skeletal fluorosis : |  |  |  |  |  |  |
|  |  |  |  | Internal control subjects (43.0 ± 14.2) |  |  |  |  |  |  |
|  |  |  |  | External control subjects (42.0 ± 15.3) |  |  |  |  |  |  |
|  |  |  |  |  |  |  |  |  |  |  |
| Drinking water, urine and epithelial cells oral mucosa |  | Cross-sectional study |  | 6 – 12 |  | 4.5 mg ⁄ L fluoride in drinking water |  | The presence of polymorphism in the COL1A2 gene was not associated with the severity of dental fluorosis. |  | Escobar-García D et al. 2016 |
|  |  |  |  |  |  |  |  |  |  |  |
| Blood |  | Cross-sectional study |  | 6 – 18 |  | Fluoride 0.72 - 0.77mg ⁄ L in Curitiba and 0.10 - 0.55mg ⁄ L in Rio de Janeiro |  | The polymorphisms rs4694075, rs5997096, and rs4970957 in AMBN, TFIP11, and TUFT1 were associated with DF dental fluorosis. |  | Küchler EC et al. 2018 |
|  |  |  |  |  |  |  |  |  |  |  |
| Drinking water, Brick tea and urine |  | Cross-sectional study |  | >16 |  | 0.04 - 4.49 mg/L fluoride in drinking water |  | Significant differences of brick-tea type fluorosis prevalence are found in four ethnic in China. |  | Liu Yang et al. 2014 |
|  |  |  |  |  |  | 2.76 mg/L fluoride in brick tea |  |  |  |  |
|  |  |  |  |  |  |  |  |  |  |  |
| Brick tea, blood and urine |  | Cross-sectional study |  | >16 |  | Brick tea type fluorosis |  | G allele of GSTP1 Rs1695 might be a protective factor for brick tea type skeletal fluorosis. |  | Wu J et al. 2015 |
|  |  |  |  |  |  |  |  |  |  |  |
| Blood |  | Cross-sectional study |  | 52.0±16.4 |  | 0.254±0.144 mg/L fluoride in drinking water |  | The elevated activity of MPO induced by endemic fluorosis in mechanism may be connected to the stimulating expression of MPO mRNA and the changed gene polymorphism. |  | Zhang T et al. 2013 |
|  |  |  |  |  |  |  |  |  |  |  |
| Blood |  | Cross-sectional study |  | 10 – 12 |  | 1.40 mg/L fluoride in drinking water |  | COMT polymorphism may increase the susceptibility to the deficits in IQ due to fluoride exposure. |  | Zhang S et al. 2015 |
|  |  |  |  |  |  |  |  |  |  |  |
| Drinking water, urine and blood |  | Cross-sectional study |  | 60 – 86 |  | Drinking water type fluorosis |  | High fluoride exposure is a potential risk factor for cognitive impairment. |  | Li M et al.2016 |
|  |  |  |  |  |  |  |  |  |  |  |
| Drinking water, and blood |  | Cross-sectional study |  | 40 – 75 |  | 0.84±0.26 mg/L,1.55±0.22 mg/L, 2.49±0.30 mg/L, and 4.06±1.15 mg/L fluoride in drinking water |  | The risk of essential hypertension in adults grows in a concentration-dependent manner of fluoride. |  | Sun L et al. 2013 |
|  |  |  |  |  |  |  |  |  |  |  |
| Drinking water and blood |  | Cross-sectional study |  | 54.33 ± 8.40, 54.77 ± 8.39, 56.03 ± 9.68, 56.88 ± 10.22 |  | < 1.20 mg/L,1.21–2.00 mg/L, 2.01–3.00 mg/L, more than 3.01 mg/L fluoride in drinking water |  | There is a significant positive relationship between excess fluoride exposure from drinking water and prevalence of carotid artery atherosclerosis in adults living in fluoride endemic areas.The possible mechanism was associated with the oxidative stress. |  | Liu H et al. 2014 |
|  |  |  |  |  |  |  |  |  |  |  |
| Blood, urine and kidney biopsies |  | Case-control study |  | 4 – 12 |  | Urine fluoride concentration (4.01± 1.83 ppm) |  | Fluoride interferes with renal anatomy and physiology, which may lead to renal pathogenesis. |  | Quadri JA et al. 2018 |
|  |  |  |  |  |  |  |  |  |  |  |
| Blood and urine |  | Cross-sectional study |  | 18-48 |  | >1.0 mg/L fluoride in drinking water |  | Fluoride affects the hypothalamus–pituitary–ovary axis hormone secretion. |  | Zhao MX et al. 2015 |
|  |  |  |  |  |  |  |  |  |  |  |
| Blood |  | Cross-sectional study |  | >18 |  | Sodium fluoride, fluorosilicic acid and sodium fluorosilicate (0.7–1.2 ppm) in drinking water |  | Fluoride added to tap water was significantly associated with increases in both the incidence and prevalence of diabetes. |  | Fluegge K et al. 2016 |
|  |  |  |  |  |  |  |  |  |  |  |
| Drinking water |  | Case-control study |  | 0-14 |  | Fluoride in drinking water in the Newfoundland and Labrador provinces of Canada. |  | Higher concentrations of fluoride in drinking water were associated with a higher incidence of childhood-onset type 1 diabetes. |  | Chafe R et al. 2017 |
|  |  |  |  |  |  |  |  |  |  |  |

| **Table 2. Summary of animal experiments on the pathogenesis of fluorosis in five years** | | | | | | | | | | |
| --- | --- | --- | --- | --- | --- | --- | --- | --- | --- | --- |
| **Animal Species** |  | **Tissue** |  | **Dose of fluoride** |  | **Fluoride-induced stress pathways, signaling or apoptosis pathways** |  | **Regulation of gene expression by fluoride exposure.** |  | **References** |
| Male SD rats(4-week-old ) |  | Incisor teeth |  | 10mg / kg NaF for 5 weeks |  | Caspase-9-Caspase-3-Bax-Bcl-2 cell-death pathway |  | **­**Caspase-9, Caspase-3, Bax;↓Bcl-2. |  | Li W et al.2017 |
| SD rats (6-week-old) and C57BL/6 mice (6-week-old) |  | Incisor teeth |  | 0, 50, 100, or 125 ppm NaF for 6 weeks |  | Oxidative stress, SIRT1/autophagy. |  | **­**ROS, SIRT1, JNK. |  | Suzuki M et al.2015 |
| Wistar male rats (6-week-old) |  | Blood and bone |  | 0, 10, 20 mg fluoride/kg/day for 1 month, 2 months and 3 months |  | Endoplasmic reticulum (ER) stress, PERK/Nrf2 signals |  | **­**Bip, XBP1, PERK, ATF4, CHOP, Nrf2; ↓ATF6. |  | Sun F et al. 2014 |
| SD male rats |  | long bones (femoral and tibiofibula) and blood |  | 100 mg/L NaF for 2 months |  | CaSR-PTH- PTHrP signals |  | **­**GaSR, RANKL, OPG; ↓PTH, PTHrp. |  | Wang Y et al. 2015 |
| Wistar male rats (weighing 150–180 g) |  | Pancreas and blood |  | 0, 10, 20 mg F-/kg day for one month |  | Insulin and the activity of osteoblasts |  | **­**ALP; ↓HbA1c. |  | Yang C et al. 2015 |
| Male SD rats (weighing 70–90 g) |  | Blood and distal femur joints |  | 100 mgF ion/L (from NaF) for 2 month, 4 months and 6 months |  | The ratio of osteoprotegerin ligand (OPGL) to osteoprotegerin (OPG) |  | **­**OPGL /OPG. |  | Yu J et al. 2013 |
| Wistar rats (one-month-old, female:male = 1:1) |  | Femur and tibiae |  | 150 mg /L NaF for 60 or 120 days |  | COL1A1 and COL1A2 in the bone tissues |  | **↓**COL1A1, COL1A2, COL I. |  | Yan X et al. 2015 |
| Kunming mice (weighting 20–25 g) |  | Hippocampus |  | 25, 50, and 100 mg/L NaF for 60 days |  | Neurotrophy and neuron adhesion |  | **­**PSD, MAG; ↓PLP, CREB, BDNF, NCAM. |  | Niu R et al.2018 |
| Male Wistar albino rats (12-week-old) |  | Brain |  | 100 mg/L NaF for 8 weeks |  | DNA damage |  | ↑ DNA damage. |  | Wang C et al. 2018 |
| Weaned female SD rats |  | Hippocampus |  | 100 mg/L NaF for three months, and 14 and 28 days old offspring were obtained as experimental subjects. |  | Histological alterations in the brain |  | No change in DCX and p38. |  | Zigu Z et al. 2017 |
| SD rats (weighing 90 – 120 g, female:male = 1:1) |  | Hippocampus and cerebellum |  | 0.5, 10, 50 mg/L NaF for 6 months |  | Mitochondrial dynamics morphology and distribution |  | **­**Fis1, Drp1; ↓Mfn1. |  | Lou DD et al. 2013 |
| SD rats with one month-old |  | Brain |  | 50 ppm NaF for 6 months, and 1, 7, 14, 21 and 28 days old offspring were obtained as experimental subjects. |  | The excitotoxic effect of modified NMDARs |  | **­**GluN1, GluN2B; ↓ GluN3A. |  | Wei N et al. 2018 |
| ICR female mice |  | Hippocampus |  | 0, 25, 50, 100 mg/L NaF, from pregnant day 7 to lactational day 21 |  | miR-124, miR-132 |  | **­**miR-124, miR-132, DGCR8; ↓ MeCP2, CREB. |  | Wang J et al. 2018 |
| Maternal Kunming mice (weighing 20–25 g) |  | Hippocampus |  | 0, 25, 50, and 100 mg/L NaF during gestation and lactation, and 21 days old offspring were obtained as experimental subjects. |  | Glutamate receptor |  | **↓**GluR2, mGluR2, NR2A, NR2B. |  | Sun Z et al. 2018 |
| Female Kunming mice (3-week-old) |  | Livers and blood |  | 100 mg/L NaF for 70 days |  | Oxidative stress |  | **­** ROS, MDA, T -NO, NOS2; ↓SOD,GSH-Px,T-AOC,Cat, GSH-Px1,SOD1. |  | Zhou BH et al. 2015 |
| Female Wistar rats (weighing 60–80 g) |  | Livers, kidneys and blood |  | 100 ppm NaF for 12 weeks |  | Oxidative stress |  | **­**MDA**,** Bax; ↓SOD,GSH-Px, Bcl-2. |  | Zhang Z et al. 2014 |
| Male SD rats (weighing 90 ± 10 g) |  | Livers and blood |  | 0, 50, 100, and 200 mg/L NaF for 120 days |  | Caspase-mediated pathways |  | **­**caspase-3 and −9. |  | Song GH et al. 2015 |
| Bufo gargarizans larvae |  | Livers |  | 42.4 mg F-/L for 0, 24, 48 and 72 h at Gosner stage 37 |  | Lipid metabolism; Oxidative stress |  | **­**HSP90, BCLAF1, TRα, TRβ; ↓ACC-1, FAE-1, SCP-2, CPT-1, SOD, GPx. |  | Bo X et al. 2018 |
| SD rats (weighing 80–100 g) |  | Livers |  | 17 mg/kg Fluorine-containing corn |  | PI3K-Akt1 signal pathway |  | **­**PI3K , Akt1 |  | Fan B et al. 2015 |
| ICR mice (four-week-old ) |  | Kidneys |  | 0, 12, 24 and 48 mg/kg body weight for 42 days |  | Oxidative stress |  | **­**ROS, MDA,PC; ↓SOD, GSH-Px, CAT, GSH. |  | Luo Q et al. 2017 |
| ICR mice (four-week-old ) |  | Kidneys |  | 0, 12, 24 and 48 mg/kg body weight for 42 days |  | NF-κB signaling pathway; Inflammatory responses |  | **­**NF-κB, p-NF-κB, NO, PGE2, iNOS, COX-2, TNF-α, IL-1β, IL-6, IL-8; ↓IκB, IL-4, IL-10. |  | Luo Q et al. 2017 |
| Male SD rats (six-week-old ) |  | Kidneys |  | 0, 75, and 150 ppm NaF in drinking water for 2 weeks |  | M2 macrophage-TGF-β1 fibroblast/myofibroblast-collagen synthesis pathway |  | **­**collagen type I,α-SMA, ED1, ED2, ED3, TGF-β1. |  | Kido T et al. 2017 |
| Male ICR mice (three-week-old ) |  | Sperm from the cauda epididymis and vas deferens |  | 0, 25, 50 and 100 mg/L NaF in drinking water for 60 days |  | mtDNA |  | **­**sperm mtDNA copy number; ↓nDNA integrity |  | Sun Z et al. 2017 |
| Male SD rats |  | Testes |  | 20, 40, 60 mg/kg fluoride-containing standard chows for 120 days |  | Oxidative Stress-mediated JNK and ERK signaling pathway |  | **­**JNK, p-ERK |  | Tian Y et al. 2017 |
| Male Kunming mice (eight-week-old) |  | Testes |  | 50 and 100 mg/L NaF in drinking water for 56 days |  | IL-17 signal pathway |  | **­**IL-17RA, IL-17RC, MAP2K1, MAP2K2, MAP2K3, MAPKAPK2 |  | Huo M et al. 2016 |
| Female Kunming mice (30-day-old ) |  | Uteri tissues from the F1 generation |  | 0, 50, 100, 150 mg /L F in drinking water for 90 days |  | MMP-9/TIMP-1 system |  | ↑MMP-9, TIMP-1. |  | Wang HW et al. 2017 |
| KM strain mice female (aged 4–6 weeks) and male (aged~ 7~8 weeks) |  | Ovaries |  | 50, 100, 150, 200mg/L NaF for 5 weeks |  | Fertilization potential of mature oocytes |  | **↓**Dazl, Stra8, Nobox, Sohlh1, ZP3 gene, Bmp15, Gdf9, H1oo, ZP2. |  | Yin S et al. 2015 |
| Female Kunming mice (30-day-old) |  | Ovaries |  | 100 mg /L F in drinking water for 90 days |  | Oxidative stress |  | **­**ROS, MDA, NO and iNO**S,**Bax,casepase-3，casepase-9; ↓SOD1, GSH-Px1, CAT, Bcl-2. |  | Wang HW et al. 2017 |
| Male Wistar rats (60-day-old ) |  | Gastrocnemius muscle |  | 0, 10, 50 ppm NaF in drinking water for 22 days |  | Insulin resistance |  | **↓**MDH, HSPB8, GRP78. |  | Lima Leite A et al. 2014 |
| Female SD rats (70-day-old ) |  | Blood and bone |  | 15 mg/L NaF in drinking water for 30 days |  | Insulin resistance |  | **↓**HOMA–IR |  | Lombarte M et al. 2013 |
|  | | | | | | | | | | |
| Arrows refer to increases (↑) or decreases (↓) genes regulation. | | | | |  |  |  |  |  |  |

| **Table 3. Summary of *in vitro* experiments on the pathogenesis of fluorosis in five years** | | | | | | | | | |
| --- | --- | --- | --- | --- | --- | --- | --- | --- | --- |
| **Cells type** |  | **dose of fluoride** |  | **fluoride-induced stress pathways, signaling and apoptosis pathways** |  | | **Regulation of gene expression by fluoride exposure** |  | **References** |
| Rat ameloblast HAT-7 cells |  | 0, 0.8, 1.2, 1.6, 3.2 mM NaF for 48 h |  | GRP78-CHOP-Bcl2 ERS cell-death pathway |  | | **­**GRP78, calreticulin, XBP1 and CHOP. |  | Zhang Y et al. 2016 |
| Mouse ameloblast-like cell line (LS8) |  | 0, 0.25, 0.5, 1, 2 mM NaF for either 24 or 48 h |  | Unfolded protein response; Proteinases |  | | **­**BiP, XBP1, Ire1a and ATF6;↓MMP-20 and KLK-4. |  | Wei W et al. 2013 |
| Mouse ameloblast-like cell line (LS8) |  | 0, 5 mM NaF for 6 h |  | Caspase-9-Caspase-3-Bax-Bcl-2 cell-death pathway |  | | **­**Caspase-9, Caspase-3, Bax;↓Bcl-2. |  | Li W et al.2017 |
| Mouse ameloblast-like cell line (LS8) |  | 0, 0.5, 1, 3, 5 mM NaF for 2 h or 6 h. |  | Oxidative stress, SIRT1/autophagy. |  | | **­**ROS, SIRT1 and JNK. |  | Suzuki M et al.2015 |
| Rat ameloblast HAT-7 cells |  | 0, 0.4, 0.8, 1.6, 3.2 and 6.4 mmol/L NaF for 24, 48 or 72 h |  | GRP78-caspase-12 ERS cell-death pathway |  | | **­**GRP78 and caspase-12. |  | Li J et al.2015 |
| Primary ameloblasts were isolated from the molar tooth germ of 4-day-old SD rats. |  | 0, 3.2 mM NaF for 12 to 72 h |  | The FasL signalling pathway |  | | **­**FASL, caspase-8 and caspase-3. |  | Wang L et al.2016 |
| Mouse meloblast-like cell line (LS8) |  | 0, 0.5, 1.0, 1.5, 2.0 mM NaF for either 24 or 48 h |  | p-ERK and p-JNK pathways |  | | **­**caspase-3, caspase-8, caspase-9, caspase-12, p-P38;↓p-ERK, p-JNK. |  | Zhao L et al.2016 |
| Mouse meloblast-like LS8 cells |  | 0, 2 mM NaF for 48 h |  | Phagocytosis of protein particles; Bcl-2 signals |  | | **­**LAMP1 and CD68;↓Bcl-2. |  | Yang T et al. 2014 |
| HAT-7 ameloblasts |  | 0, 0.8, 1.2, 1.6 mM/L NaF |  | Beclin1-mTOR autophagy |  | | **­**Beclin1;↓mTOR. |  | Lei S et al. 2015 |
| The human osteoblast-like cell line (Saos-2) |  | 0,0.1,0.2,0.4,0.8, nd 1.6 mM NaF for 24, 48, and 72 h |  | BMP/Smad signaling pathway |  | | **­**BGP, BALP and Smad4. |  | Huo L et al. 2013 |
| The osteoblastic cell line ( MC3T3-E1) |  | 0, 8 mg/L fluoride for 7 days |  | MicroRNAs |  | | 45 upregulated and 31 downregulated miRNAs. |  | Wang Y et al. 2017 |
| The osteoblastic cell line ( MC3T3-E1) |  | 0.5, 1.0, 2.0, 4.0, 8.0, 12.0, and 20.0 mg/L fluoride for 1, 2, 4, and 10 days |  | ER-UPR signaling pathway |  | | **­** Runx2, Bip, PERK, e-IF2α, XBP-1, ATF6, CHOP;↓ATF4. |  | Zhou YL et ai. 2013 |
| The osteoblastic cell line ( MC3T3-E1) |  | 0, 0.1, 1, 2, 4, 8, 16, 20, 32, and 64 mg/L fluoride for 1, 3, 7, and 14 days |  | ER-UPR signaling pathway |  | | **­** Bip, PERK, ATF4, Xbp1 and ATF6. |  | Li XN et al. 2014 |
| The osteoblastic cell line ( MC3T3-E1) |  | 0, 2, 8 and 20 mg/l fluoride for 2, 4,10 days |  | Endoplasmic reticulum (ER) stress, PERK/Nrf2 signals |  | | **­** PERK, Nrf2;↓ATF4, Runx2, OPG. |  | Sun F et al. 2014 |
| The osteoblastic cell line ( MC3T3-E1) |  | 0, 1, 5, 10, and 30 mg/L NaF for 24, 48, 72, and 96 h |  | Apoptosis through ROS-dependent mitochondrial signaling pathway |  | | **­**ROS levels; **↓**mitochondrial membrane potentials. |  | Yan X et al. 2017 |
| The osteoblastic cell line ( MC3T3-E1) |  | 0, 2, 5, and 10 mg/L NaF for 48 h |  | CaSR-PTH- PTHrP signals |  | | **­**GaSR, RANKL, OPG;↓PTH, PTHrp. |  | Wang Y et al. 2015 |
| The bone marrow stem cells (BMSC) were isolated from the femurs of Kunming mice |  | 0, 1,4, and 16 mg/L fluoride for 4 and 7 days |  | The activity of osteoblasts |  | | **­**ALP;↓HbA1c. |  | Yang C et al. 2015 |
| The RAW264.7 cell line |  | 0, 1,4 and 16 mg/L of NaF for 4 and 7 days |  | TβR1/Smad3 pathway |  | | **­**TβR1 and Smad3. |  | Yu H et al. 2018 |
| Primary bone marrow cells |  | 0, 0.5, 2 and 8 mg/L fluoride for 1, 3, 5 days |  | Osteoclast formation and function |  | | **↓**NFAT c1. |  | Junrui Pei et al. 2014 |
| Primary chondrocytes were isolated from arthrodial cartilage of neonatal Wistar rats |  | 0, 1.5, 2.0, 2.5, 3.0, 3.5 and 4.0 mM NaF for 24, 48 and 72 h |  | Sox9- HIF-1α pathway |  | | **­**Bax, cleaved caspase-9, -12, -3 proteins;↓Bcl-2, HIF-1α, Sox9, Col II. |  | Meng H et al.2014 |
| Osteoclasts were formed from bone marrow cells of C57BL/6 mice |  | 0, 0.5, 2, and 8 mg/L fluoride for 1, 2, 3 and 5 days |  |  |  | | **­**TRAP5b, CTR, OSCAR mRNA;↓MMP9 ,CK mRNA. |  | Junrui P et al. 2016 |
| Human osteosarcoma (HOS) cell line |  | 0, 8 mg/L NaF |  | Histone modiﬁcation |  | | ↑Histone methyltransferases (EHMT1 and EHZ2) and global histone trimethylation (H3K9 and H3K27) . |  | Daiwile AP et al. 2018 |
| The immortalized murine microglia cell line (BV-2) |  | 0, 1, 5, 10, 50, 100 mg/L NaF for 12 and 24h |  | ROS-JNK/MAPK signaling pathway |  | | **­**ROS, NO,TNF-𝛼, IL-1𝛽, p38, p-ERK and p-JNK. |  | Yan L et al.2013 |
| Human neuroblastoma (SH-SY5Y) cells |  | 0, 20, 40, 80 mg/mL NaF for 24 h; with 40 mg/L NaF for 3, 6, 12, 18, 24 h |  | Intracellular Ca21 concentration ([Ca21]i) and ROS |  | | **­**LDH, Ca^2+^, ROS. |  | Zhixia Xu et al. 2013 |
| Primary cultured neurons from SD rat embryos |  | 0.5–100 ppm NaF for 48 h |  | The excitotoxic effect of modified NMDARs |  | | **­**GluN1, GluN2B; ↓ GluN3A. |  | Wei N et al. 2018 |
| Human umbilical vein endothelial cells (HUVECs) |  | 1.2 μg/mL NaF for 24 h |  | PI3K/AKT/eNOS pathway |  | | **↓**NO, PI3K/AKT/eNOS pathway, PI3K and pAKT/AKT. |  | Huang Y et al. 2018 |
| Primary hepatocytes were isolated from Balb/c and sfx/sfx mice |  | 0, 0.25, 0.5, 1, 2, 4, and 8 mM NaF for 12, 24 and 48 h |  | Oxidative stress |  | | **­**MDA;↓SOD, GPx, and CAT |  | Wei W et al. 2014 |
| Mouse spermatozoa were collected from the cauda epididymides of 8- to 12-week old ICR mice. |  | 0, 2.5, 5, and 10 mM NaF for 90 min |  | Sperm function and fertilization |  | | **↓**Intracellular calcium concentration, protein kinase-A activity, and tyrosine phosphorylation |  | Kim J et al. 2015 |
| Oocyte were isolated from porcine ovaries. |  | 0, 30, 60,100, and 150 μg/ml NaF for 24 and 30 h |  | Oxidative stress |  | | **­** Cathepsin B activity; ↓GSH. |  | Liang S et al. 2017 |
|  |  |  |  |  |  | |  |  |  |
| Arrows refer to increases (↑) or decreases (↓) genes regulation. | | | | | |  | |  |  |
